# Supplementary figures and images for: Patterns of recent natural selection on genetic loci associated with sexually differentiated human body size and shape phenotypes
Source: PLoS Genet. 2021 Jun 3;17(6):e1009562. doi: 10.1371/journal.pgen.1009562 (PMC8174730; doi:10.1371/journal.pgen.1009562)

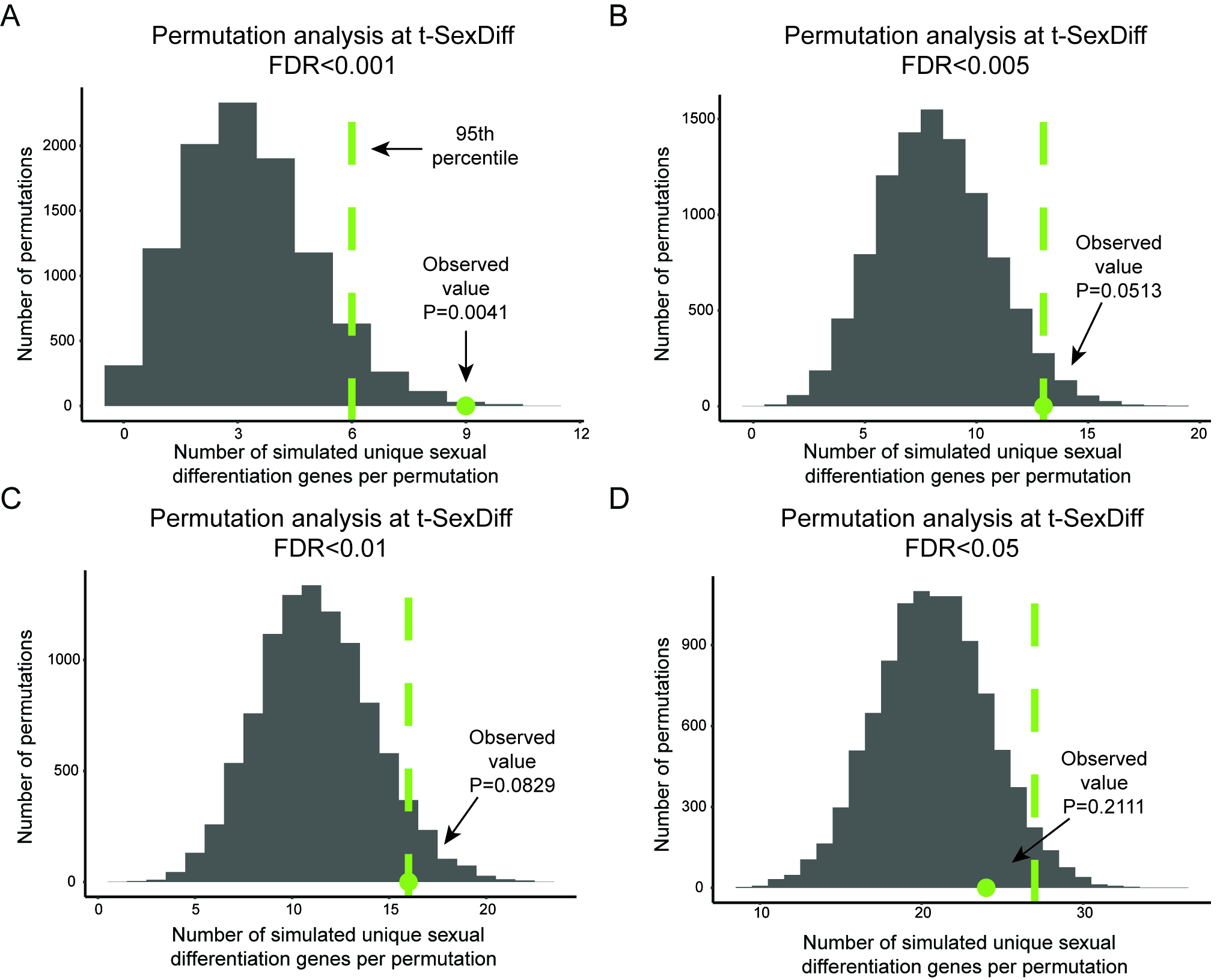

Supplement: S1 Fig — Permutation analysis of the number of genes involved in sexual differentiation for all anthropometric SNPs at every FDR threshold. Data are the frequency of distribution of our results for 10,000 permuted data sets. The empirical P-value represents the probability that the observed value of sexual differentiation genes from our SexDiff-associated SNP pool is equal to or greater than those from a randomly selected set. (TIF) [file pgen.1009562.s001.tif]

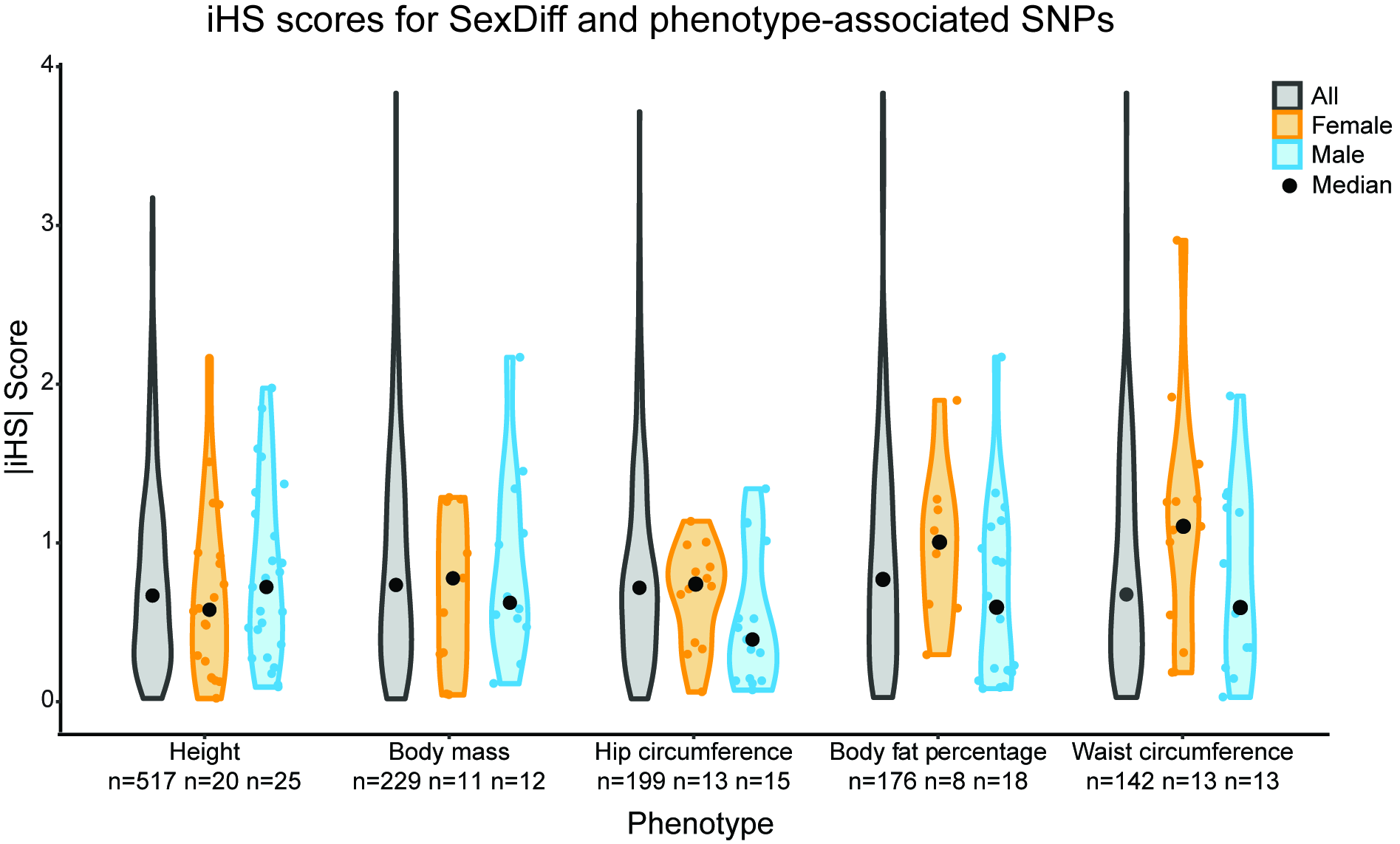

Supplement: S2 Fig — The iHS distributions for each set of female and male SexDiff pruned SNPs were compared to those for the corresponding phenotype-association set using a permutation analysis. None of the distributions were significantly different. (TIF) [file pgen.1009562.s002.tif]
