# Supplementary material for: Patterns of recent natural selection on genetic loci associated with sexually differentiated human body size and shape phenotypes
Source: PLoS Genet. 2021 Jun 3;17(6):e1009562. doi: 10.1371/journal.pgen.1009562 (PMC8174730; doi:10.1371/journal.pgen.1009562)
Supplement: S1 Table — (DOCX) [file pgen.1009562.s003.docx]

**S1 Table:** Observed number of SexDiff-associated SNPs at each FDR threshold for every phenotype.

| Phenotype | Number of phenotype-associated SNPs | Number of SexDiff-associated SNPs | | | | Ratio^a^ |
| --- | --- | --- | --- | --- | --- | --- |
|  |  | FDR  0.05 | FDR  0.01 | FDR  0.005 | FDR  0.001 |  |
| Height | 67738 | 9654 | 4242 | 3083 | 677 | 0.0010 |
| Body mass | 15669 | 3588 | 1940 | 1101 | 540 | 0.0345 |
| Hip circumference | 12580 | 3822 | 1991 | 1422 | 808 | 0.0642 |
| Body fat percentage | 10538 | 3488 | 1727 | 1034 | 439 | 0.0417 |
| Waist circumference | 7674 | 2478 | 896 | 741 | 550 | 0.0718 |

^a^Ratio of SexDiff-associated SNPs at the FDR threshold of 0.001 to the number of phenotype-associated SNPs
