# Supplementary material for: Patterns of recent natural selection on genetic loci associated with sexually differentiated human body size and shape phenotypes
Source: PLoS Genet. 2021 Jun 3;17(6):e1009562. doi: 10.1371/journal.pgen.1009562 (PMC8174730; doi:10.1371/journal.pgen.1009562)
Supplement: S2 Table — aProportion of SDG genes observed in each SNP group bRatio of proportion of SDG genes observed in the group of SexDiff-associated SNPs to the proportion of SDG genes observed in the group of non SexDiff-associated SNPs. (DOCX) [file pgen.1009562.s004.docx]

**S2 Table:** Observed unique sexual differentiation genes (SDG) and total number of genes for SexDiff-associated SNPs and Non SexDiff-associated SNPs

| FDR threshold | Number of unique sexual differentiation genes | | | | | | Ratio^b^ |
| --- | --- | --- | --- | --- | --- | --- | --- |
|  | SexDiff-associated SNPs | | | Non SexDiff-associated SNPs | | |  |
|  | SDG | Total Genes | Proportion^a^ | SDG | Total Genes | Proportion^a^ |  |
| 0.001 | 9 | 162 | 0.0556 | 52 | 2544 | 0.0204 | 2.7254 |
| 0.005 | 13 | 396 | 0.0328 | 51 | 2499 | 0.0204 | 1.6078 |
| 0.01 | 16 | 545 | 0.0294 | 49 | 2437 | 0.0201 | 1.4627 |
| 0.05 | 24 | 1005 | 0.0239 | 47 | 2269 | 0.0207 | 1.1546 |

^a^Proportion of SDG genes observed in each SNP group ^b^Ratio of proportion of SDG genes observed in the group of SexDiff-associated SNPs to the proportion of SDG genes observed in the group of non SexDiff-associated SNPs
