# Supplementary material for: Patterns of recent natural selection on genetic loci associated with sexually differentiated human body size and shape phenotypes
Source: PLoS Genet. 2021 Jun 3;17(6):e1009562. doi: 10.1371/journal.pgen.1009562 (PMC8174730; doi:10.1371/journal.pgen.1009562)
Supplement: S3 Table — aNumber of pruned SexDiff-associated SNPs at an FDR threshold of 0.001 bMean log2 ratio of female trait effect size to the male trait effect size cOne-sided t-test P-value comparing distribution of the log2(ratio) dPermutation P-value of the probability that the mean log2(ratio) could be observed by chance when compared to phenotype-associated SNPs. (DOCX) [file pgen.1009562.s005.docx]

**S3 Table:** Observed log_2_ ratio of female to male beta values and p-values for each set of Female SexDiff-associated SNPs

| Phenotype | #SNPs^a^ | Mean  log_2_(ratio)^b^ | P-value to zero^c^ | FDR to zero | P-value to phenotype-associated SNPs^d^ | FDR to phenotype-associated SNPs |
| --- | --- | --- | --- | --- | --- | --- |
| Height | 21 | 2.5244 | 4.8x10^-10^ | 4.8x10^-9^ | <0.001 | 0.001 |
| Body mass | 11 | 2.7726 | 9.8x10^-7^ | 1.6x10^-6^ | <0.001 | 0.001 |
| Hip circumference | 13 | 3.2853 | 1.9x10^-6^ | 2.7x10^-6^ | <0.001 | 0.001 |
| Body fat percentage | 9 | 3.2393 | 3.3x10^-5^ | 3.3x10^-5^ | <0.001 | 0.001 |
| Waist circumference | 14 | 3.3511 | 5.1x10^-6^ | 6.4x10^-6^ | <0.001 | 0.001 |

^a^Number of pruned SexDiff-associated SNPs at an FDR threshold of 0.001 ^b^Mean log_2_ ratio of female trait effect size to the male trait effect size ^c^One-sided t-test P-value comparing distribution of the log_2_(ratio) ^d^Permutation P-value of the probability that the mean log_2_(ratio) could be observed by chance when compared to phenotype-associated SNPs
