# Supplementary material for: Patterns of recent natural selection on genetic loci associated with sexually differentiated human body size and shape phenotypes
Source: PLoS Genet. 2021 Jun 3;17(6):e1009562. doi: 10.1371/journal.pgen.1009562 (PMC8174730; doi:10.1371/journal.pgen.1009562)
Supplement: S4 Table — aNumber of pruned SexDiff-associated SNPs at an FDR threshold of 0.001 bMean log2 ratio of female trait effect size to the male trait effect size cOne-sided t-test P-value comparing distribution of the log2(ratio) dPermutation P-value of the probability that the mean log2(ratio) could be observed by chance when compared to phenotype-associated SNPs. (DOCX) [file pgen.1009562.s006.docx]

**S4 Table:** Observed log_2_ ratio of female to male beta values and p-values for each set of Male SexDiff-associated SNPs

| Phenotype | #SNPs^a^ | Mean  log_2_(ratio)^b^ | P-value to zero^c^ | FDR to zero | P-value to phenotype-associated SNPs^d^ | FDR to phenotype-associated SNPs |
| --- | --- | --- | --- | --- | --- | --- |
| Height | 25 | -2.2579 | 2.3x10^-7^ | 5.8x10^-7^ | <0.001 | 0.001 |
| Body mass | 12 | -2.1049 | 2.2x10^-7^ | 5.8x10^-7^ | <0.001 | 0.001 |
| Hip circumference | 15 | -2.9211 | 7.8x10^-8^ | 3.9x10^-7^ | <0.001 | 0.001 |
| Body fat percentage | 18 | -3.3821 | 8.0x10^-7^ | 1.6x10^-6^ | <0.001 | 0.001 |
| Waist circumference | 13 | -4.2544 | 1.8x10^-5^ | 2.0x10^-5^ | <0.001 | 0.001 |

^a^Number of pruned SexDiff-associated SNPs at an FDR threshold of 0.001 ^b^Mean log_2_ ratio of female trait effect size to the male trait effect size ^c^One-sided t-test P-value comparing distribution of the log_2_(ratio) ^d^Permutation P-value of the probability that the mean log_2_(ratio) could be observed by chance when compared to phenotype-associated SNPs
