# Supplementary material for: Patterns of recent natural selection on genetic loci associated with sexually differentiated human body size and shape phenotypes
Source: PLoS Genet. 2021 Jun 3;17(6):e1009562. doi: 10.1371/journal.pgen.1009562 (PMC8174730; doi:10.1371/journal.pgen.1009562)
Supplement: S5 Table — aNumber of pruned SexDiff-associated SNPs at an FDR threshold of 0.001 bAverage trait-SDS score of pruned set of SexDiff-associated SNPs cPermutation P-value of the probability that the trait-SDS score for each sex could be observed by chance when compared to phenotype-associated SNPs. (DOCX) [file pgen.1009562.s007.docx]

**S5 Table:** Observed trait-SDS and permutation P-values for each set of Female SexDiff-associated SNPs and Male SexDiff-associated SNPs permuted against phenotype-associated SNPs.

| Phenotype | Female | | | | Male | | | |
| --- | --- | --- | --- | --- | --- | --- | --- | --- |
|  | #SNPs^a^ | trait-SDS^b^ | P-value to phenotype-associated SNPs^c^ | FDR | #SNPs^a^ | trait-SDS^b^ | P-value to phenotype-associated SNPs^c^ | FDR |
| Height | 21 | 0.2403 | 0.6608 | 0.8246 | 25 | 0.0540 | 0.6944 | 0.8246 |
| Body mass | 11 | 0.2331 | 0.7864 | 0.8246 | 12 | 0.2359 | 0.7408 | 0.8246 |
| Hip circumference | 13 | 0.1739 | 0.4688 | 0.8246 | 15 | 0.7068 | 0.1208 | 0.6040 |
| Body fat percentage | 9 | **0.8272** | **0.0038** | **0.0380** | 18 | 0.1146 | 0.3046 | 0.8246 |
| Waist circumference | 14 | 0.0222 | 0.8246 | 0.8246 | 13 | 0.2161 | 0.6040 | 0.8246 |

^a^Number of pruned SexDiff-associated SNPs at an FDR threshold of 0.001 ^b^Average trait-SDS score of pruned set of SexDiff-associated SNPs ^c^Permutation P-value of the probability that the trait-SDS score for each sex could be observed by chance when compared to phenotype-associated SNPs -
