# Supplementary material for: Patterns of recent natural selection on genetic loci associated with sexually differentiated human body size and shape phenotypes
Source: PLoS Genet. 2021 Jun 3;17(6):e1009562. doi: 10.1371/journal.pgen.1009562 (PMC8174730; doi:10.1371/journal.pgen.1009562)
Supplement: S7 Table — aNumber of pruned SexDiff-associated SNPs at an FDR threshold of 0.001 bAverage |iHS| score of pruned set of SexDiff-associated SNPs cPermutation P-value of the probability that the |iHS| score for each sex could be observed by chance when compared to phenotype-associated SNPs. (DOCX) [file pgen.1009562.s009.docx]

**S7 Table:** Observed average |iHS| scores and permutation P-values for each set of Female SexDiff-associated SNPs and Male SexDiff-associated SNPs permuted against phenotype-associated SNPs.

| Phenotype | Female | | | | Male | | | |
| --- | --- | --- | --- | --- | --- | --- | --- | --- |
|  | #SNPs^a^ | \|iHS\| | P-value to phenotype-associated SNPs^c^ | FDR | #SNPs^a^ | \|iHS\| | P-value to phenotype-associated SNPs^c^ | FDR |
| Height | 20 | 0.6586 | 0.7989 | 0.989 | 25 | 0.7822 | 0.4291 | 0.989 |
| Body mass | 11 | 0.6705 | 0.8368 | 0.989 | 12 | 0.8284 | 0.5659 | 0.989 |
| Hip circumference | 13 | 0.6547 | 0.8333 | 0.989 | 15 | 0.4975 | 0.9890 | 0.989 |
| Body fat percentage | 8 | 0.9729 | 0.3266 | 0.989 | 18 | 0.6570 | 0.9321 | 0.989 |
| Waist circumference | 13 | 1.1015 | 0.0770 | 0.770 | 13 | 0.7548 | 0.6384 | 0.989 |

^a^Number of pruned SexDiff-associated SNPs at an FDR threshold of 0.001 ^b^Average |iHS| score of pruned set of SexDiff-associated SNPs ^c^Permutation P-value of the probability that the |iHS| score for each sex could be observed by chance when compared to phenotype-associated SNPs
