# Supplementary material for: Patterns of recent natural selection on genetic loci associated with sexually differentiated human body size and shape phenotypes
Source: PLoS Genet. 2021 Jun 3;17(6):e1009562. doi: 10.1371/journal.pgen.1009562 (PMC8174730; doi:10.1371/journal.pgen.1009562)
Supplement: S8 Table — aNumber of pruned SexDiff-associated SNPs at an FDR threshold of 0.001 bPermutation P-value of the probability that the number of intergenic SNPs in each set of SexDiff-associated SNPs could be observed by chance when compared to phenotype-associated SNPs. (DOCX) [file pgen.1009562.s010.docx]

**S8 Table:** Observed number of intergenic SNPs and permutation P-values for each set of Female SexDiff-associated SNPs and Male SexDiff-associated SNPs permuted against phenotype- associated SNPs.

| Phenotype | Female | | | | Male | | | |
| --- | --- | --- | --- | --- | --- | --- | --- | --- |
|  | #SNPs^a^ | #Inter-genic SNPs | P-value to phenotype-associated SNPs^b^ | FDR | #SNPs^a^ | #Inter-genic SNPs | P-value to phenotype-associated SNPs^b^ | FDR |
| Height | 21 | 9 | 0.1498 | 0.2497 | 25 | 11 | 0.0998 | 0.2497 |
| Body mass | 11 | 5 | 0.1168 | 0.2497 | 12 | 4 | 0.4978 | 0.5531 |
| Hip circumference | 13 | 4 | 0.6204 | 0.6204 | 15 | 8 | 0.0178 | 0.0890 |
| Body fat percentage | 9 | 4 | 0.2312 | 0.2890 | 18 | 8 | 0.2186 | 0.2890 |
| Waist circumference | 14 | 6 | 0.1470 | 0.2497 | 13 | 8 | 0.0048 | 0.0480 |

^a^Number of pruned SexDiff-associated SNPs at an FDR threshold of 0.001 ^b^Permutation P-value of the probability that the number of intergenic SNPs in each set of SexDiff-associated SNPs could be observed by chance when compared to phenotype-associated SNPs
