# Supplementary material for: Patterns of recent natural selection on genetic loci associated with sexually differentiated human body size and shape phenotypes
Source: PLoS Genet. 2021 Jun 3;17(6):e1009562. doi: 10.1371/journal.pgen.1009562 (PMC8174730; doi:10.1371/journal.pgen.1009562)
Supplement: S9 Table — aAs referred to in the Neale lab manifest released on July 31, 2018 b Correlation for each phenotype calculated as the Spearman rank correlation coefficient between beta values of men and women. (DOCX) [file pgen.1009562.s011.docx]

**S9 Table:** Phenotype information

| Phenotype | Phenotype description^a^ | Code^a^ | Sample sizes | | rho^b^ |
| --- | --- | --- | --- | --- | --- |
|  |  |  | # Females | #Males |  |
| Height | standing height | 50_irnt | 193,785 | 166,603 | 0.44617 |
| Body mass | weight | 21002_irnt | 193,627 | 166,489 | 0.37463 |
| Hip circumference | hip circumference | 49_irnt | 193,814 | 166,707 | 0.33003 |
| Body fat percentage | body fat percentage | 23099_irnt | 190,991 | 163,637 | 0.34575 |
| Waist circumference | waist circumference | 48_irnt | 193,828 | 166,736 | 0.31999 |

^a^As referred to in the Neale lab manifest released on July 31, 2018 ^b^ Correlation for each phenotype calculated as the Spearman rank correlation coefficient between beta values of females and males
