# Supplementary material for: Patterns of recent natural selection on genetic loci associated with sexually differentiated human body size and shape phenotypes
Source: PLoS Genet. 2021 Jun 3;17(6):e1009562. doi: 10.1371/journal.pgen.1009562 (PMC8174730; doi:10.1371/journal.pgen.1009562)
Supplement: S10 Table — (DOCX) [file pgen.1009562.s012.docx]

**S10 Table:** Observed trait-SDS for each set of pruned phenotype-associated SNP groups.

| Phenotype | # Pruned SNPs | tSDS |
| --- | --- | --- |
| Height | 532 | 0.1325 |
| Body mass | 239 | 0.1496 |
| Hip circumference | 210 | 0.2123 |
| Body fat percentage | 181 | -0.1244 |
| Waist circumference | 147 | 0.0784 |
