# Supplementary material for: Patterns of recent natural selection on genetic loci associated with sexually differentiated human body size and shape phenotypes
Source: PLoS Genet. 2021 Jun 3;17(6):e1009562. doi: 10.1371/journal.pgen.1009562 (PMC8174730; doi:10.1371/journal.pgen.1009562)
Supplement: Attachment — Submitted filename: Arner et al Response to Reviewers.docx [file pgen.1009562.s013.docx]

We would like to thank all the reviewers for their positive and encouraging reviews. We have addressed the requested revisions as described below in our point-by-point responses. All reviewer comments are in bold below, followed by our responses including with relevant text from the manuscript in italics. Alterations to manuscript are underlined. In the manuscript itself, updated text is indicated in red font.

**Reviewer 1:**

**The manuscript by Arner et al. tests whether genetic variants are associated with sexually differentiated phenotypes in humans using UKBioBank data. The authors first find SNPs associated with different traits in the UK Biobank. They then test whether the strength of the association differs between males and females. Indeed, the authors find that these SexDiff SNPs are enriched in genes involved in sexual differentiation. Lastly, the authors test whether the SexDiff SNPs show greater signals of recent positive selection, as compared to SNPs associated with the overall phenotype. The authors find that alleles associated with increased body fat percentage in only females tend to show signals of positive selection via the SDS statistic more so than SNPs associated with body fat percentage. The other traits examined do not show signals of such sexually differentiated positive selection.**

**Overall this paper addresses an interesting question that has not received as much attention in the empirical human genetics literature. The statistical analyses seem solid and carefully thought-out. I have several comments to help improve the manuscript:**

**1) Starting on line 154 forward: In this section, the authors test whether the SexDiff SNPs are enriched in genes that have a known role in sexual differentiation. This is a nice addition to the paper and helps strengthen the case that these SNPs are biologically functional. Were the associated SNPs pruned for linkage disequilibrium for these analyses? It was unclear to me at which stage of the analyses the LD pruning was done. Unless I’m missing something (please correct me if I am), the LD pruning should be done prior to testing for enrichments as the enrichment tests are assuming that the SNPs are more or less independent. Further, as genes in different functional categories may have different recombination rates, patterns of LD, etc, it makes sense to include putatively independent SNPs as much as possible.**

Response -- We agree with the reviewer that careful consideration of LD is critical when performing enrichment analyses. In this particular analysis, instead of counting and analyzing the number of SexDiff-associated SNPs in each gene, which could overinflate enrichment results due to LD, we counted and analyzed the number of *genes* that contain or are nearby one or more SexDiff-associated SNPs. We updated this section of the manuscript to help make this more clear:

*For each t-SexDiff FDR threshold, we determined the number of unique sexual differentiation (GO:0007548) genes with one or more co-localized SexDiff-associated SNPs (combined across the five phenotypes in our study). We similarly identified the number of all genes (of those in the GO database) that were co-localized with at least one SexDiff-associated SNP and calculated the proportion of the number of GO:0007548 genes to the total number of genes. We conducted the same analysis for the set of SNPs associated with the phenotypes in general but not associated with sexual differentiation. Finally, we estimated the GO:0007548 enrichment ratio for SexDiff-associated to non SexDiff-associated SNPs by dividing the two proportions. By focusing this analysis on counts of genes rather than counts of SNPs, we limit potential linkage disequilibrium (LD)-based enrichment inflation. That is, a gene would only be counted once regardless of how many SexDiff-associated SNPs are located within or nearby that gene.*

For subsequent analyses that were not gene based (e.g. sex-specific effect size ratios and signatures of positive selection), we did use an aggressive pruning strategy in which we limited our analyses to a maximum of one SNP per each of 1,703 approximately LD-independent blocks of the human genome.

**2) Figure 2 and associated analyses: I appreciate the resampling approach to test for enrichment. Was there a specific reason why the permutation analysis was used instead of Fisher’s exact test? It might be good to clarify more on this.**

Response -- Great point, and this is something that we should have clarified explicitly (and now we have done so, in our revision). As mentioned above, for this analysis we identified the number of unique genes that contained or were nearby one or more SexDiff-associated SNPs, rather than the total number of SNPs in the gene regions. Although our sets of SNPs are independent (SexDiff-associated SNPs and non-SexDiff SNPs that were trait-associated), the two sets of SNPs may still be in the same genes. For example, a SexDiff-associated SNPs may be in the same gene as a non-SexDiff-associated SNP (indeed there are such cases), which would violate the assumptions of the Fisher’s exact test. We have updated our manuscript to now include a sentence regarding the violation of the test’s assumptions to the paragraph explaining our permutations:

*To test whether the proportion of GO:0007548 genes co-localized with at least one SexDiff-associated SNP was significantly greater than expected by chance, we used a simple permutation scheme. From the set of 2,191 total GO classified genes that were co-localized with at least one phenotype-associated SNP, we randomly selected the same number of total genes that were co-localized with one or more SexDiff-associated SNPs at the FDR cutoff being considered (e.g. 135 genes at FDR<0.001) and counted the number of GO:0007548 genes represented. We repeated this process 10,000 times for each FDR cutoff and compared the resulting distributions to the actual number of GO:0007548 genes co-localized with at least one SexDiff-associated SNP to calculate empirical P-values. We used this permutation-based approach rather than a Fisher’s exact test because some genes were co-localized with both SexDiff-associated SNPs and non-SexDiff phenotype-associated SNPs. The observed proportion of GO:0007548 genes is very unlikely to be explained by chance at the most stringent FDR<0.001 threshold (P=0.0041;* ***Figure 2B****), with increasing probability for the FDR thresholds of 0.005, 0.01, and 0.05 (P=0.0513, P=0.0829, and P=0.2111, respectively; Figure S1).*

**3) Section starting on line 222: I had a hard time understanding the statistical or biological relevance of this section. What is the purpose of testing for whether the SexDiff variants are disproportionately associated with greater phenotype effect sizes in the sex with the stronger association values? Does this not occur by design? Better motivating the purpose of this analysis would strengthen the manuscript in my view.**

Response -- Thank you for this comment. Interestingly, we had added this analysis only after receiving a comment from a peer who pointed out that theoretically (even if unlikely) our p-value-based approach could lead to the identification of SNPs with lower variance with trait values but not necessarily greater effect sizes. Thus, we performed this analysis to demonstrate that our going-in assumptions were being met. In our revised manuscript, we have updated the text introduction of this section to be more explicit on this point.

*Our statistical approach technically identifies genetic variants with significantly different strengths of association for a given trait between males and females. Before proceeding, we sought to confirm that these identified variants are disproportionately associated with greater phenotype effect sizes in the sex with the stronger association value, and not merely with lower trait value variance.*

**4) Figure 3 and tSDS analyses: For each trait, the authors compare the tSDS statistic distribution of the sexDiff SNPs to the tSDS statistic distribution for SNPs associated with the trait. Is this the right null distribution? What if the traits themselves are undergoing positive selection, just not in a sexually differentiated way? Then the SexDiff SNPs may not be expected to look different even if they are under selection too. Additionally, it’s not clear to me what the ideal null distribution for comparison would be in these cases. More explanation as to the rationale for the choice of the null set of SNPs would be good.**

Response -- Thank you for this point. After careful thought we do believe that the set of pruned phenotype-associated SNPs we used in this analysis is the correct null distribution for the signatures of selection that we hoped to identify. Specifically, we do want to evaluate signatures of selection on SNPs associated with sexual differentiation significantly beyond any such signatures on the underlying trait. We have added the following text to help clarify this point:

*To examine the evolutionary histories of alleles associated with anthropometric trait sex differences, we aimed to determine whether any signatures of positive selection for SexDiff-associated SNPs differed in either magnitude or direction from any such signatures for alleles associated with the phenotypes (but not necessarily sex differences) themselves. For this analysis, we again considered the sets of LD-pruned SexDiff-associated SNPs divided into those that are disproportionately associated with trait variation in females vs. those disproportionately associated with trait variation in males for each phenotype. Using a permutation analysis, we tested whether the average trait-SDS values for the pruned female or male SexDiff-associated SNPs were significantly different than the value for the corresponding pruned set of all phenotype-associated SNPs (****Figure 3B;*** *Table S6). For example, there were n=21 pruned SexDiff-associated SNPs disproportionately associated with female height and n=532 pruned SNPs associated with height generally (but not necessarily with sex differences). We randomly selected 21 of the 532 pruned height-associated SNPs and calculated the average trait-SDS score. We repeated this process 10,000 times and calculated an empirical P-value based on the proportion (multiplied by two; see Methods) of permuted observations with equal or more extreme average trait-SDS values than the observed average trait-SDS for the actual female height SexDiff-associated SNPs (average trait-SDS=0.240; P=0.66). This test was then repeated for the male SexDiff-associated SNPs for height, and the female and male SexDiff-associated SNPs for each of the remaining four anthropometric traits.*

**5) Figure 3 and tSDS analyses: Are the control SNPs for the tSDS statistic matched for allele frequency and recombination rate to the SexDiff SNPs in each comparison? It would be important to match for these putative confounders that differ across the genome and may influence the tSDS values.**

Response -- Thank you for suggesting this analysis, which we have now conducted and report in the revised version of our manuscript. Specifically, in each iteration of our permutations we matched each SexDiff-associated SNP with a phenotype-associated SNPs within +/- 0.05 minor allele frequency of the SexDiff-associated SNP. This resulted in the same finding. We have updated our manuscript (Results and Methods) as follows and we also included a supplementary table to report these findings:

*Results: To examine the evolutionary histories of alleles associated with anthropometric trait sex differences, we again considered the sets of LD-pruned SexDiff-associated SNPs divided into those that are disproportionately associated with trait variation in females vs. those disproportionately associated with trait variation in males for each phenotype. Using a permutation analysis, we tested whether the average trait-SDS values for the pruned female or male SexDiff-associated SNPs were significantly different than the value for the corresponding pruned set of all phenotype-associated SNPs (****Figure 3B;*** *Table S5). For example, there were n=21 pruned SexDiff-associated SNPs disproportionately associated with female height and n=532 pruned SNPs associated with height generally (but not necessarily with sex differences). We randomly selected 21 of the 532 pruned height-associated SNPs and calculated the average trait-SDS score. We repeated this process 10,000 times and calculated an empirical P-value based on the proportion (multiplied by two; see Methods) of permuted observations with equal or more extreme average trait-SDS values than the observed average trait-SDS for the actual female height SexDiff-associated SNPs (average trait-SDS=0.240; P=0.66). This test was then repeated for the male SexDiff-associated SNPs for height, and the female and male SexDiff-associated SNPs for each of the remaining four anthropometric traits. We also performed a similar permutation but with the additional step of matching minor allele frequencies (+/- 0.05) between the original set of SexDiff-associated SNPs and the set of phenotype-associated SNPs selected in each permutation. The pattern of results was unchanged relative to the above (Table S6).*

*Methods: We also performed trait-SDS permutation analysis with minor allele frequency (MAF) matching between SexDiff and phenotype-associated SNPs. Specifically, for each SexDiff-associated SNP we identified all phenotype-associated SNPs with +/- 0.05 MAF. We then randomly selected one of these SNPs for inclusion in the permuted dataset and removed it from our list of phenotype-associated SNPs to be drawn from in the MAF matching to other SexDiff-associated SNPs so that it wouldn’t be represented twice in the same permutation. We repeated this procedure 10,000 times, randomizing the input order of SexDiff-associated SNPs each time.*

**Table S6:** Observed trait-SDS and permutation P-values for each set of Female SexDiff-associated SNPs and Male SexDiff-associated SNPs permuted against phenotype-associated SNPs matched for minor allele frequency.

^a^Number of pruned SexDiff-associated SNPs at an FDR threshold of 0.001 ^b^Average trait-SDS score of pruned set of SexDiff-associated SNPs ^c^Permutation P-value of the probability that the trait-SDS score for each sex could be observed by chance when compared to phenotype-associated SNPs matched for minor allele frequency

**6) Discussion: The authors included a nice discussion about population stratification as a potential confounder. I think it would be good to also discuss whether associative mating might be expected to influence any of these results. Further, on the other side, it would also be good to include some sort of discussion of the power of these analyses. It appears the authors made a number of conservative decisions in their statistical analyses (which I think is good, unless otherwise noted here) and it would be good to put this into context. For example, how strongly do the negative results reject the hypotheses of recent reductions in anthropometric trait sex differences in agricultural societies?**

Response -- These are good suggestions. In our revised manuscript, we now discuss several points related to the power of our analyses. Ultimately, we decided not to discuss assortative mating because it is unclear how assortative mating would affect the selection statistic results (beyond the potential role of assortative mating in the evolutionary processes themselves):

*We note that we made a number of conservative choices (for example, with aggressive pruning to account for linkage disequilibrium) in our analytical approach, meaning that our failure to reject the null hypothesis for each of these four traits should not be interpreted as evidence that no selection on them occurred. Still, even with our conservative analytical approach we did find evidence that the average frequencies of alleles disproportionately associated with greater female body fat percentage significantly increased over the past 3,000 years, a pattern consistent with polygenic adaptation. Given that females have higher average body fat percentages than men in historic and contemporary populations, the direction of polygenic adaptation in the population we studied would actually be opposite to expectations under hypotheses of recent adaptive reductions in anthropometric trait sex differences in agricultural societies. However, since SNPs can be pleiotropically associated with multiple phenotypes (32), we cannot definitively conclude that positive selection acted directly on female body fat percentage. Regardless, at the very least we did not find positive support for the prevailing hypothesis concerning the evolution of sex differences in recent human evolution.*

**Minor comments:**

**1. Lines 36-38: This wording was a little confusing. Maybe say something like “SNPs associated with significant differences in association between males and females are enriched genes with known roles in sexual differentiation, as compared to other SNPs associated with the trait”.**

Response -- As mentioned above, our enrichment analyses were set up to identify the number of genes with SexDiff-SNPs rather than the number of SNPs in genes in order to account for LD. Therefore, after careful thought we have decided that we would prefer to maintain our current wording for this sentence.

**2. Line 63: This seems like an odd start to the paper. Why not just start talking about “sexually differentiated traits” and their biology, rather than say that these are not “sexually dimorphic”. That statement can come elsewhere later in the Intro.**

Response -- Thank you for this suggestion. We have restructured the introduction paragraph to first discuss sexually differentiated traits as follows:

*In many vertebrate species, it is not uncommon for morphological phenotypes to have average size and shape differences between females and males (1). Traits with average phenotype values that differ by sex but with overlapping trait distributions – such as human height and body fat percentage – are described as ‘sexually differentiated’ traits. Technically, the more commonly-used term ‘sexually dimorphic’ is specific to non-overlapping traits; for example, exaggerated ornamentation in male guppies, peacocks, and mandrills (2–5).*

**3. Lines 137: It would be good to give a little more context and description of the t-SexDiff test here. I realize that it’s fully explained in the Methods, but given that Results come first, I think it would be nice to give a short explanation to those who do not read the Methods.**

Response -- We are grateful for this suggestion and have added a brief explanation of the t-SexDiff test. The section referenced now reads:

*From the sets of phenotype-associated SNPs that were significant in females, males, or both for each phenotype (SexDiff-associated SNPs), we identified those SNPs with significant differences in the statistical strengths of association for the female vs. male-specific GWAS results, using the t-SexDiff statistic (19). This statistic estimates the probability of difference between female-specific and male-specific effect sizes given their standard errors and considering the genome-wide correlation between male and female effect sizes for each trait (see Methods). To account for multiple testing, we performed this analysis with four different false-discovery rate (FDR) cutoffs for each phenotype: 0.05, 0.01, 0.005, and 0.001.*

**4). Lines 142-144: Is this a ratio or a proportion?**

Response – Thank you for noticing this imprecision, we indeed meant a proportion, here. We have corrected the sentence as follows:

*In addition to identifying SexDiff-associated SNPs, for each phenotype we calculated the proportion of SexDiff-associated SNPs at the FDR threshold of 0.001 to the number of phenotype-associated SNPs, which ranged from 0.0010 for height to 0.0718 for waist circumference.*

**5) Enrichment analysis in Figure 2: Were 162 or 135 genes selected? Both seem to be used (lines 191 and 206).**

Response – Thank you for catching this error. We have corrected the number of genes in line 191.

*From the set of 2,191 total GO classified genes that were co-localized with at least one phenotype-associated SNP, we randomly selected the same number of total genes that were co-localized with one or more SexDiff-associated SNPs at the FDR cutoff being considered (e.g. 162 genes at FDR<0.001) and counted the number of GO:0007548 genes represented.*

**6) Figure 2B: To avoid confusion, the “differentiation” label on the x-axis might better be called “Number of GO:00007548 genes per permutation” or something like that. I worry that some may think “differentiation” might refer to the Fst or the population genetic version of differentiation, which of course is not what’s meant.**

Response -- We are grateful for this suggestion, which we have taken. The Figure 2B x-axis label is now:

*Number of GO:00007548 genes per permutation*

**7) Line 293: Specify how many tests were corrected for with the FDR adjustment.**

Response – We corrected for ten tests with the FDR adjustment and updated line 293 to include this information.

*For nine of the comparisons, trait-SDS distributions for the sets of pruned SexDiff-associated SNPs were not significantly different (following FDR adjustment for ten tests) from those for SNPs associated with the corresponding general phenotype (****Figure 3B****; Table S5).*

**Reviewer #2:**

**In this manuscript the authors are interested in two related questions: whether there is evidence of significant differential genetic architecture between human males and females across multiple complex traits and, if so, whether these differences can be explained by recent positive selection in the directions expected based on the first set of results. The authors are interested in this question in part due to anthropological theory that states the change in human history to subsistence farming from hunter and gatherers should be accompanied by a reduction in differentiating characteristics between males and females, as the necessary traits in males and females become more homogenous.**

**The authors approach these questions by analyzing white British individuals in the UK BioBank dataset across multiple complex traits. They conduct sex-differentiated genome-wide association studies and compare both the number of associations between the sexes and the strength of association. They also look at whether genes related to sexual differentiation are enriched among their top hits. Lastly, using their sex-differentiated association SNPs (sex-diff SNPs), they conduct positive selection analyses using the singleton density score, a method that can identify recent positive selection.**

**The authors find evidence for both differential genetic architecture between males and females across the traits analyzed, as well as evidence for at least one trait and sex combination (body fat percentage in females) to be undergoing recent positive selection, though in the opposite direction (increasing in allele frequency) than one would expect from the anthropological theory.**

**Overall I think this is a good manuscript and study design. I think the analyses and justifications make sense, and the flow of the different parts work well. I also appreciate how the work shown here has a connection to a theory from anthropology, grounding the results as well as potentially broadening their impact too. I do have a few suggestions regarding some additional analyses that might further bolster the positive selection sections, as well as a few minor comments.**

Response: Thank you!

**Major Comments:**

**1) For the SDS results, I am wondering whether the presence of older positive selection might affect the results somehow, or at least change our interpretation of the results. For instance, if we find that there is evidence for older positive selection on body fat percentage in females, then maybe our interpretation of the SDS results changes from recent selection due to the change in subsistence strategy to simply ongoing selection for sexual differentiation. In fact maybe we would expect some amount of sexual differentiation to be continuously selected for due to phenomena like assortative mating or sexual conflict. To get at this, could the authors run some other tests for positive selection, such as Berg and Coop’s Qs or statistics for selective sweeps? If there is evidence for older positive selection on the traits that did not show significant SDS results as well, I would wonder then if it’s possible that SDS might be less effective due to presumably lower levels of local genetic variation.**

Response -- Thank you for asking this question; it is a good and interesting point. Accordingly, we performed an additional analysis with the integrated haplotype score (iHS) statistic. We selected this instead of the Berg and Coop Qx statistic because of the time horizon of the analysis and because like tSDS, iHS is a within-population analysis. We have updated our manuscript to include this analysis as follows:

Results -- *While our hypothesis testing framework is focused primarily on analyses of recent signatures of positive natural selection, we also investigated whether the sets of LD-pruned SexDiff-associated SNPs were enriched for signatures of relatively more ancient positive selection. To do so we conducted an identical permutation analysis as above with the trait-SDS values, but instead using absolute values of the integrated haplotype score (iHS) statistic, a within-population haplotype-based statistic that can be used to identify putative signatures of positive selection from across the past ~25,000 years (21). We used previously-published iHS data (22) computed for the Great Britain population (GBR) from the 1000 Genomes Project (23). Absolute iHS values were not significantly different for any of the sets of SexDiff-associated SNPs relative to the corresponding phenotype-associated SNPs (Figure S2, Table S7).*

Methods -- *For our iHS-based analysis,* *we used a database of genome-wide SNP iHS scores that were previously computed (22) using data from the 1000 Genomes project (23). We specifically used iHS data from the Great Britain (GBR) population due to population similarity with our GWAS data. We considered the absolute value of the standardized iHS for each SNP, and we removed SNPs from our dataset that did not have a corresponding iHS score. To estimate the probability that the average iHS score for each trait and sex could be observed by chance given the distribution of iHS scores for phenotype-associated SNPs, we used a permutation scheme identical to that used for the primary trait-SDS analysis (the version without consideration of minor allele frequencies).*

Citations added:

40. Voight BF, Kudaravalli S, Wen X, Pritchard JK. A Map of Recent Positive Selection in the Human Genome. Hurst L, editor. PLoS Biol. 2006 Mar 7;4(3):e72.

41. Johnson KE, Voight BF. Patterns of shared signatures of recent positive selection across human populations. Nat Ecol Evol. 2018 Apr 19;2(4):713–20.

42. Auton A, Abecasis GR, Altshuler DM, Durbin RM, Bentley DR, Chakravarti A, et al. A global reference for human genetic variation. Vol. 526, Nature. Nature Publishing Group; 2015. p. 68–74.

**Figure S2.** Sex-specific iHS scores for anthropometric SexDiff and phenotype-associated SNPs. The iHS distributions for each set of female and male SexDiff pruned SNPs were compared to those for the corresponding phenotype-association set using a permutation analysis. None of the distributions were significantly different.

**Table S7:** Observed |iHS| and permutation P-values for each set of Female SexDiff-associated SNPs and Male SexDiff-associated SNPs permuted against phenotype-associated SNPs.

^a^Number of pruned SexDiff-associated SNPs at an FDR threshold of 0.001 ^b^Average |iHS| score of pruned set of SexDiff-associated SNPs ^c^Permutation P-value of the probability that the |iHS| score for each sex could be observed by chance when compared to phenotype-associated SNPs

**2) Similarly for the SDS results, is there any difference between the distribution of sex-diff SNPs and the non-sex-diff SNPs in terms of genic vs. intergenic regions? Once again I am wondering about other factors that may be affecting the results, and whether if lower levels of local genetic variation for the sex-diff SNPs might somehow impact the SDS results, ie if sex-diff SNPs were somehow more often in genic regions than non-sex-diff SNPs are. If this were the case then maybe some of the negative SDS results for the other traits are a result of being under powered.**

Response -- Thank you for suggesting this analysis. We ran a permutation analysis to test whether there was a genic-intergenic difference between each of our ten sets of SexDiff-associated SNPs and the corresponding phenotype-associated SNPs. None of the ten sets of SexDiff-associated SNPs were more often in genic regions than the corresponding phenotype-associated SNPs were. However, we did find that male SexDiff-associated SNPs significantly associated with hip circumference were more likely to be found in intergenic than genic regions when compared to the corresponding phenotype-associated SNPs. We have updated our manuscript to include this analysis as follows:

Results text: *Finally, because positive selection may occur more often within or nearby genic regions of the genome (40), we also tested whether our sets of pruned SexDiff-associated SNPs and corresponding phenotype-associated SNPs were unevenly distributed among genic (annotated genes +/- 10,000 bp) and intergenic regions. None of the ten sets of SexDiff-associated SNPs were enriched for genic regions. However, we found that SNPs significantly associated with male hip circumference variation were more likely to be intergenic relative to general hip circumference-associated loci (Table S8).*

*Methods text: To test whether there were significant differences in the distributions of pruned SexDiff-associated SNPs and corresponding pruned phenotype-associated SNPs, we determined whether or not each SNP was located within +/- 10,000 bp of a gene annotated in the Gene Ontology database. We compared the ratio of genic:intergenic SNPs for each of our ten sets of SexDiff-associated and corresponding phenotype -associated SNPs for each trait using a permutation analysis (Table S8). For each set of SexDiff-associated SNPs, we randomly selected the same number of SNPs from the corresponding set of phenotype-associated SNPs and counted the number of SNPs in intergenic regions. We then repeated this procedure 10,000 times and computed an empirical P-value as the proportion of permuted data sets with an equal or greater to number of SNPs in intergenic regions when compared to our observation for the associated set of SexDiff-associated SNPs. This proportion was then multiplied by two to account for the two-tailed test (i.e. the number of intergenic SexDiff-associated SNPs could have been significantly greater than or less than that of phenotype-associated SNPs). We computed FDR values to account for multiple testing.*

***Table S7:*** *Observed number of intergenic SNPs and permutation P-values for each set of Female SexDiff-associated SNPs and Male SexDiff-associated SNPs permutated against phenotype-associated SNPs.*

^a^Number of pruned SexDiff-associated SNPs at an FDR threshold of 0.001 ^b^Permutation P-value of the probability that the number of intergenic SNPs in each set of SexDiff-associated SNPs could be observed by chance when compared to phenotype-associated SNPs

Citations added:

40. Voight BF, Kudaravalli S, Wen X, Pritchard JK. A Map of Recent Positive Selection in the Human Genome. Hurst L, editor. PLoS Biol. 2006 Mar 7;4(3):e72.

**3) The authors mention the possibility that pleiotropy could be occurring among the sex-diff SNPs showing evidence for positive selection in female body fat percentage. Since the authors have already used the summary statistics from the Ben Neale UKB GWAS results, I would be curious whether as a group these SNPs are also all associated with any other traits. If as a group we find a pattern of traits these SNPs are associated with, or on the other hand find no evidence these SNPs are associated with any other traits (at least among the traits previously analyzed), that could be an interesting additional piece of information to help with the interpretation.**

Response -- Thank you for suggesting this analysis, which has led us to a very beneficial finding regarding biological insight and the overall biological plausibility of our results! We used the GWAS Catalog to identify other phenotypes each LD-pruned SexDiff and Phenotype-associated SNP is associated with, with supplementary data tables for the full results. We noticed a large number of SexDiff SNPs that were also “sex hormone binding globulin level” trait-associated SNPs. We used a permutation analysis to confirm that the co-occurrence of SexDiff- and sex hormone binding globulin level-associated SNPs is greater than expected, relative to the co-occurrence observed with all Phenotype-associated SNPs. We have updated our manuscript as follows:

Results: *In a second assessment of the biological plausibility of our results, we queried the GWAS Catalog (34) to identify any pleiotropic co-occurrence between our SexDiff-associated (FDR<0.001) or phenotype-associated SNPs and significant associations for all other GWAS Catalog traits. To avoid linkage disequilibrium (LD)-based result inflation of co-occurrence, we selected a maximum of one SNP (zero if no SNPs in the region were significantly associated with a trait) with the lowest t-SexDiff p-value across any of the five studied traits for each of 1,703 approximately LD-independent blocks of the human genome (35). Of the 693 total SNPs in this curated dataset, 117 SNPs (17%) were significantly associated with sex differences for at least one of the five traits, with the remaining 576 SNPs (83%) associated with one or more of the five phenotypes but not sex differences.*

*Upon reviewing the overlap with the curated set of SexDiff-associated SNPs and the GWAS Catalog trait associations, we immediately noticed “Sex hormone binding globulin level” annotations (36–38) for 3 of the 117 SexDiff-associated SNPs (2.6%), including the two SNPs with the most extreme t-SexDiff p-values (****Fig. 2C****). In contrast, sex hormone binding globulin level associations were annotated for 4 or the 576 phenotype but not SexDiff-associated SNPs (0.7%; Fisher’s exact test; P=0.098). We also used a permutation analysis to estimate the probability of observing 3 SNPs with both SexDiff- and sex hormone binding globulin level-associations, given the occurrence rate for all loci in this curated SNP subset (P=0.063;* ***Fig. 2D****).*

Methods: *We studied pleiotropic relationships between our anthropometric SexDiff and phenotype traits and other traits with the aid of the GWAS Catalog (35; accessed 18 Feb 2021;). After concatenating all SexDiff (FDR<0.001) and phenotype-associated SNPs from across the five studied traits, we pruned this total set of SNPs to a maximum of one SNP per each of approximately 1,703 approximately LD-independent blocks of the human genome (37). For each block, we specifically chose the one SNP with the lowest t-SexDiff p-value (if a block did not have any significant phenotype-associated SNPs for any trait, zero SNPs were included from that block). There were 693 total SNPs in this curated dataset, 117 of which (17%) were significantly associated with sex differences for at least one of the 5 traits, with 576 (83%) significantly associated with one or more of the five phenotypes but not sex differences. After reviewing the full results (data to be included in the Dryad Digital Repository Deposition), we used a permutation analysis to estimate the probability that SexDiff-associated SNPs were more likely than expected by chance to also be associated with the “sex hormone binding globulin level” trait. Specifically, from our curated set of 693 SNPs, we randomly selected the number of SNPs equal to the number of observed SexDiff-associated SNPs (117) and counted how many were also associated with sex hormone binding globulin level as per the GWAS catalog. We repeated this procedure 10,000 times and computed the proportion of permuted data sets with equal or more extreme numbers of sex hormone binding globulin level-associated SNPs compared to the result for the SexDiff-associated SNPs.*

Citations added:

34. Buniello A, MacArthur JAL, Cerezo M, Harris LW, Hayhurst J, Malangone C, et al. The NHGRI-EBI GWAS Catalog of published genome-wide association studies, targeted arrays and summary statistics 2019. Nucleic Acids Res. 2019;47(D1):D1005–12.

36.Ruth KS, Day FR, Tyrrell J, Thompson DJ, Wood AR, Mahajan A, et al. Using human genetics to understand the disease impacts of testosterone in men and women. Nat Med. 2020 Feb 10;26(2):252–8.

37. Prescott J, Thompson DJ, Kraft P, Chanock SJ, Audley T, Brown J, et al. Genome-Wide Association Study of Circulating Estradiol, Testosterone, and Sex Hormone-Binding Globulin in Postmenopausal Women. Gorlova OY, editor. PLoS One. 2012 Jun 4;7(6):e37815.

38. Coviello AD, Haring R, Wellons M, Vaidya D, Lehtimäki T, Keildson S, et al. A Genome-Wide Association Meta-Analysis of Circulating Sex Hormone–Binding Globulin Reveals Multiple Loci Implicated in Sex Steroid Hormone Regulation. Gibson G, editor. PLoS Genet. 2012 Jul 19;8(7):e1002805.

***Figure 2. Co-localization of SexDiff-associated SNPs and loci with known roles in sexual differentiation****. (A) For each t-SexDiff FDR threshold, we computed the proportion of the number of genes in the “Sexual Differentiation” Gene Ontology category (GO:0007548) to the number of all Gene Ontology genes with at least one co-localized SexDiff-associated SNP (+/- 10,000 base pairs). We also computed the same proportion for the set of SNPs significantly associated with our studied phenotypes in general but not with sexual differentiation. Values shown are ratios of these two proportions at each t-SexDiff FDR threshold. The green line indicates the 1:1 ratio that would be expected in the absence of any disproportionate co-localization between SexDiff-associated SNPs and GO:00007548 genes. (B) Permutation analysis of the number of genes involved in sexual differentiation co-localized with at least one SexDiff-associated SNPs at our most stringent FDR threshold (q<0.001). From the set of 2,191 total GO-classified genes that were co-localized with at least one phenotype-associated SNP, we randomly selected 162 genes, the number of total genes that were co-localized with one or more SexDiff-associated SNPs at the FDR<0.001 cutoff. Of these 162 genes, we counted and recorded the number of GO:0007548 genes represented. We repeated this process 10,000 times and computed an empirical p-value (P=0.0041) as the proportion of permutations with a greater than or equal number of GO:0007548 genes as the observed value for FDR<0.001 SexDiff-associated SNPs (9 genes). Results for similar analyses based on SexDiff FDR thresholds 0.005, 0.01, and 0.05 are shown in Figure S1. (C) Manhattan plot depicting the -log10 values for the t-SexDiff statistic for a curated set of LD-pruned SexDiff and phenotype-associated SNPs. Specifically, we selected a maximum of one SNP with the lowest t-SexDiff p-value across any of the five studied phenotypes from each of 1,703 approximately LD-independent blocks of the human genome. Yellow dots indicate SexDiff-associated SNPs at an FDR of 0.001 (for at least one of the five traits), and pink dots indicate phenotype-associated SNPs that did not cross the FDR=0.001 threshold for any trait. SNPs outlined in black are also significantly associated with the sex hormone binding globulin levels as annotated in the GWAS catalog. (D) From the curated set of 693 total LD-pruned SexDiff and phenotype-associated SNPs, we randomly selected 117 SNPs (the number of pruned SexDiff-associated SNPs) and counted how many were also associated with sex hormone globulin blinding levels. We repeated this process 10,000 times and computed an empirical p-value of P=0.0626.*

**Minor Comments:**

**1) For Figure 1 I have a few comments, mostly related to consistency. I don’t believe the red vs. pink motif in 1A continues throughout the rest of the figure (likely due to the combination of red and yellow later on), so I’m not sure if it should be used at all. The Bonferroni p-value threshold line should be labeled in 1A and 1B since the FDR lines are labeled later on as well. And in the legend the FDR lines are described as ‘green bars’, though in the copy of the manuscript I have, the bars are blue. This might just be my copy, but it should be checked and corrected if needed.**

Response -- Thank you for pointing out the inconsistency between panels of Figure 1. We have changed the colors of 1A to dark and light orange and the colors of 1B to dark and light blue to denote alternating even/odd chromosomes. In 1C and 1D we have replicated this color scheme, again with dark orange and blue SNPs denoting odd chromosomes and light orange and blue SNPs denoting even chromosomes. We also changed the FDR lines to shades of gray. To account for this color change, we have also changed the color of the violin plots in Figure 3.

**2) For lines 233-235, I think “...we prepared similar LD-pruned sets of all SNPs significantly associated with the phenotype for each of the five traits…” would be more clear if it included something such as “...sets of all SNPs significantly associated with the phenotype, /irregardless of sex differentiation or not/,...”. Right now it’s unclear if this comparison set of SNPs includes those that are sex-diff or not. It becomes a bit more clear later on through the analyses, but explicitly delineating this here might help.**

Response – Thank you for this suggestion – we were insufficiently clear. We have clarified this sentence as follows:

*For comparison to the disproportionate female and male SexDiff-associated SNPs, we prepared similar LD-pruned sets of all SNPs significantly associated with the phenotype, regardless of their associations with sex differences, for each of the five traits.*

**3) There are a few sentences that are awkwardly constructed. I would recommend revising them**.

Response – Thank you for pointing out these sentences. We have revised each sentence as follows:

**a) Lines 75-77: “/Large ranges in the degree of body size and shape sexually differentiated traits/ are repeatedly observed among…” (unclear here)**

*Finally, there are major differences in the degree of body size and shape sex differences between closely related species, suggesting the potential for the relatively rapid evolution of sexually differentiated traits.*

**b) Lines 101-104: “/With the recent availability of a greatly increased participant sample size, this analysis represents a powerful extension of the several previous GWAS-based approaches/ that have studied the genetic architecture of…’ (feels clunky)**

*With a very large number of UK Biobank participants (~361,000 individuals in the dataset we analyzed), this analysis represents a powerful extension of several previous GWAS-based studies of the genetic architecture of human sexually differentiated anthropometric traits.*

**c) Lines 278-281: “...for the corresponding pruned set of all phenotype-associated SNPs (Figure 3B; Table S6), /using a permutation analysis/” (maybe start the sentence with this?)**

*Using a permutation analysis, we tested whether the average trait-SDS values for the pruned female or male SexDiff-associated SNPs were significantly different than the value for the corresponding pruned set of all phenotype-associated SNPs (Figure 3B; Table S5).*

**4) This may not be necessary for the paper, but could the authors explain what the concern with BMI is? The paper cited has this at the end of its conclusion: “ABSI expresses the excess risk from high WC in a convenient form that is complementary to BMI and to other known risk factors.” Is this mostly a reflection of body shape being a better complementary phenotype to traits such as waist and hip circumference, whereas BMI may be too correlated? If so, the authors may want to be more explicit with their reasoning beyond ‘concerns about this metric’ -- this makes it seem like BMI is a potentially ‘bad metric’, which is maybe not the intention here.**

Response -- Thank you for this point. We chose not to include BMI because of concerns that the metric does not accurately indicate obesity and obesity-related health risks and for other ethical reasons. However, we did include both height and body mass, the input variables of BMI, in our set of sexually differentiated phenotypes. We have added an expanded explanation as follows:

*We did not include body mass index (BMI) in our set of body size and shape phenotypes following concerns that have been voiced about this metric related to its failure to quantify body shape when indicating obesity and obesity-related health risks (26-27) and other, ethical issues (28). We instead included the two constituents of BMI (height and body mass) as independent variables in our analysis.*

Citations added:

26. Gómez-Ambrosi J, Silva C, Galofré JC, Escalada J, Santos S, Millán D, et al. Body mass index classification misses subjects with increased cardiometabolic risk factors related to elevated adiposity. Int J Obes. 2012 Feb 17;36(2):286–94.

27. Nevill AM, Stewart AD, Olds T, Holder R. Relationship between adiposity and body size reveals limitations of BMI. Am J Phys Anthropol. 2006 Jan;129(1):151–6.

28. Humphreys S. The unethical use of BMI in contemporary general practice. Br J Gen Pract. 2010 Sep;60(578):696–7.
